# A scoping review: virtual patients for communication skills in medical undergraduates

**DOI:** 10.1186/s12909-022-03474-9

**Published:** 2022-06-03

**Authors:** Síle Kelly, Erica Smyth, Paul Murphy, Teresa Pawlikowska

**Affiliations:** 1 Department of Medicine, RCSI University of Medicine and Health Science, Smurfit Building, ERC, Beaumont Hospital, Beaumont Road, Dublin 9, Ireland; 2 Health Professions Education Centre, RCSI University of Medicine and Health Science, Dublin, Ireland; 3 Library, RCSI University of Medicine and Health Science, Dublin, Ireland; 4 Health Professions Education Centre, RCSI University of Medicine and Health Science, Dublin, Ireland

**Keywords:** Virtual Patients, Technology enhanced learning, Communication skills, Undergraduate, Virtual learning, Online medical teaching, Consultation skills, Medical students

## Abstract

**Background:**

Communication is an essential competence for medical students. Virtual patients (VP), computerized educational tools where users take the role of doctor, are increasingly used. Despite the wide range of VP utilization, evidence-based practical guidance on supporting development of communication skills for medical students remains unclear. We focused this scoping review on VP affordance for student learning especially important in the current environment of constrained patient access.

**Methods:**

This scoping review followed Arksey & O’Malley’s methodology. We tested and used a search strategy involving six databases, resulting in 5,262 citations. Two reviewers independently screened titles, full texts (*n*= 158) and finally performed data extraction on fifty-five included articles. To support consideration of educational affordance the authors employed a pragmatic framework (derived from activity theory) to map included studies on VP structure, curricular alignment, mediation of VP activity, and socio-cultural context.

**Results:**

Findings suggest that not only the VP itself, but also its contextualization and associated curricular activities influence outcomes. The VP was trialled in the highest proportion of papers as a one-off intervention (19 studies), for an average duration of 44.9 minutes (range 10-120min), mainly in senior medical students (*n*=23), notably the largest group of studies did not have VP activities with explicit curricular integration (47%). There was relatively little repeated practice, low levels of feedback, self-reflection, and assessment. Students viewed VPs overall, citing authenticity and ease of use as important features. Resource implications are often omitted, and costings would facilitate a more complete understanding of implications of VP use.

**Conclusion:**

Students should be provided with maximal opportunity to draw out the VPs’ full potential through repeated practice, without time-constraint and with curricular alignment. Feedback delivery enabling reflection and mastery is also key. The authors recommend educators to explicitly balance computerized authenticity with instructional design integrated within the curriculum.

**Supplementary Information:**

The online version contains supplementary material available at 10.1186/s12909-022-03474-9.

## Background

In the healthcare setting, communication skills are fundamental to clinical practice. With an expected 120,000-200,000 consultations across the span of a career, the effective physician communicator is vital to the delivery of high-quality patient care, improved patient satisfaction, and enhanced health outcomes for both patient and doctor [1]. In the US, medical error ranks in the top ten causes of death, with 70% of preventable medical errors rooted in communication error before the sentinel event occurs [2].

The Association of American Medical Colleges (AAMC, 2005) [3] and many other medical regulatory bodies [4, 5] now recognise the value of communication skills development and the CanMeds Framework has supported a shift towards domains of competency based medical education (CBE) [6, 7]. From the perspective of the innumerable benefits, and avoidable risk to patients, educational interventions for communication skills are an essential component in undergraduate medicine [8–10].

### Virtual patients

Personalised computer usage has grown exponentially and few sectors are left untouched [11, 12]. This growth is mirrored in medical education by Technology Enhanced Learning (TEL) [13], however its use in medical education remains poorly understood, and research in this area often fails to inform practice [13, 14].

Virtual patients (VP) are computer simulations allowing learners to take on the role of the healthcare professional for the purpose of developing skill and knowledge in a particular area [15]. Theoretically the VP offers advantages over human standardised patients, as learners can practice skills at their convenience, learner-set pace and individualised interactivity: all importantly without putting patients at risk [2, 16]. The lack of standardisation in the clinical setting can give rise to unequal learning among medical students [17] and development of these vital skills should not be left to chance [8, 18, 19].

### COVID-19 (SARS- CoV-2) pandemic

The COVID-19 pandemic underscores the importance of patient-physician communication in maintaining trust, partnership and quality in the delivery of patient care. The impact of SARS-CoV-2 on the delivery of healthcare education has led to many schools seeking a technological solution for the development of essential competencies, while keeping students and patients safe. Several recent BEME reviews highlighted the pivot to virtual environments for clinical learning [20]. One of the major challenges of this dramatic shift was the lack of standardised telemedicine curricula [20]. It was recommended that educators must underpin these new developments with theory, as well utilise the full potential of available technologies, rather than to simply replace traditional methods with technology [21]. ‘Virtual Patients’ have the potential to be the technological support and curricular adjunct to meet these ongoing challenges of virtual education [15].

### Aim

Given the diversity of approaches in this field, we selected a scoping review to explore “What is known about virtual patients used to support the development of medical undergraduate communication skills?” our aim being to build on educational innovation and identify gaps in this sphere.

## Methods

To develop this review and maximize educational utility we conducted a pilot search within two databases (PubMed and Embase) to investigate heterogeneity and capture (estimated at 80%), revealing mostly definitional issues concerning virtual patients for medical students’ communication skills training.

‘Communication skills’ are a complex set of inextricably-linked processes interfacing with a host of patient-physician interactions. We adopted a broad approach and included papers that covered at least a component of communication skills e.g. information gathering. The technology of ‘virtual patients’ advanced before terminology [ 22, 23], giving rise to issues of nomenclature. In addition, researchers apply ‘VP’ to other digital technologies such as simulated patient manikins, second-life avatars, and even non-digital learning tools [ 23, 24].

We selected the American Association of Medical Colleges (AAMC) definition of virtual patients first outlined in 2006 [25] to be as inclusive as possible incorporating a large variety of learning technologies, while remaining refined enough to exclude those studies that are likely to be irrelevant [26] a VP is *“A specific type of computer-based program that simulates real-life clinical scenarios; learners emulate the roles of health care providers to obtain a history, conduct a physical exam and make diagnostic and therapeutic decisions”* [25]*.*

The methodological choice was informed by the STORIES statement with intention to produce a rigorous review that benefits the wider healthcare community of this expanding field [27]. We used the Arksey & O’Malley (see Supplementary Appendix 1) [28], scoping review methodology which is divided into five parts: 1. Identifying the research question; 2. Identifying relevant studies; 3. Study selection; 4. Charting the data; and 5. Collating, summarising and reporting results [28, 29].

We developed the review question iteratively, informed by the pilot study and discussions with computer scientists working in this and related fields. The scoping review was framed to ensure a broad sweep of literature in order to maximize utility for this educational development.

### Identifying relevant studies

We searched six electronic databases (PubMed, EMBASE, PychInfo, Web of Science, ERIC, CINAHL), and iteratively tested search strings to achieve maximal inclusion developed in consultation with an experienced research librarian (P.M.). To supplement the search, we scanned the reference lists of studies for papers fulfilling inclusion criteria.

### Study selection

We included peer-reviewed journal research papers with a component of communication skills educational interventions, undergraduate medical students and virtual patients as defined by the AAMC [25]. We excluded non-English language papers due to limited resources, and also opinion pieces, commentaries, and conference abstracts without full paper publications, studies that focussed on interventions with manikins, human standardised patients, and systems requiring specialised hardware not readily available. We had no restrictions on year of publication. Quality criteria were not assessed, as this is not a part of scoping review methodology [28].

The main researcher (S.K.) reviewed title and abstracts applying inclusion and exclusion criteria in tranches that were independently reviewed (by TP). In the event of uncertainty articles were discussed as a research team and conflicts resolved by consensus (*n*=107).

### Charting the data

We used the pilot search to develop and test a framework used for data extraction, which would capture relevant themes, enable us to map them and also be useful for educational development.

#### Kirkpatrick framework

The Kirkpatrick framework (see Supplementary Appendix 2) has been widely used to evaluate the impact of educational interventions [30]. However, it has also been critiqued as being restrictive for a complex field such as medical education and not having provision to evaluate all outcomes that may be derived from the rich variety of methodologies used in health professions education [31]. We used the Kirkpatrick framework, as it is a common language affording familiarity for the community of educators, however, in exploring our pilot data it became evident that a more dynamic relational lens would provide the necessary granularity.

#### Activity theory framework for virtual patients

Ellaway & Davies’ [32] adaptation of Leont’ev and Egenström’s ‘Activity Theory’ provides this in moving away from the reductionist lens of asking whether a Virtual Patient ‘works’ or not, towards pragmatic enquiry into how VPs are used or could be used [32, 33]. We have adopted the five interconnected domains of virtual patient scholarship for this scoping review: 1. Objectives: how virtual patients align to a curriculum or program; 2. Actions: what learners and teachers do around VPs; 3. Operations: the clicks and key presses needed to make VPs run; 4. Mediation of VP activity; and 5. Socio-cultural context for VP activity.

#### Collecting, summarising and reporting results

We developed a data abstraction form (see Supplementary Appendix 3) through an iterative process, which built upon the above frameworks, allowing us to focus on practical aspects of studies and identify gaps. It was tested independently by S.K. and T.P. Disagreements at any stage were resolved through discussion and consensus. Inter-rater agreement was assessed using Cohen’s kappa statistic. We report the results in tabular and narrative forms below.

## Results

**Fig. 1:**
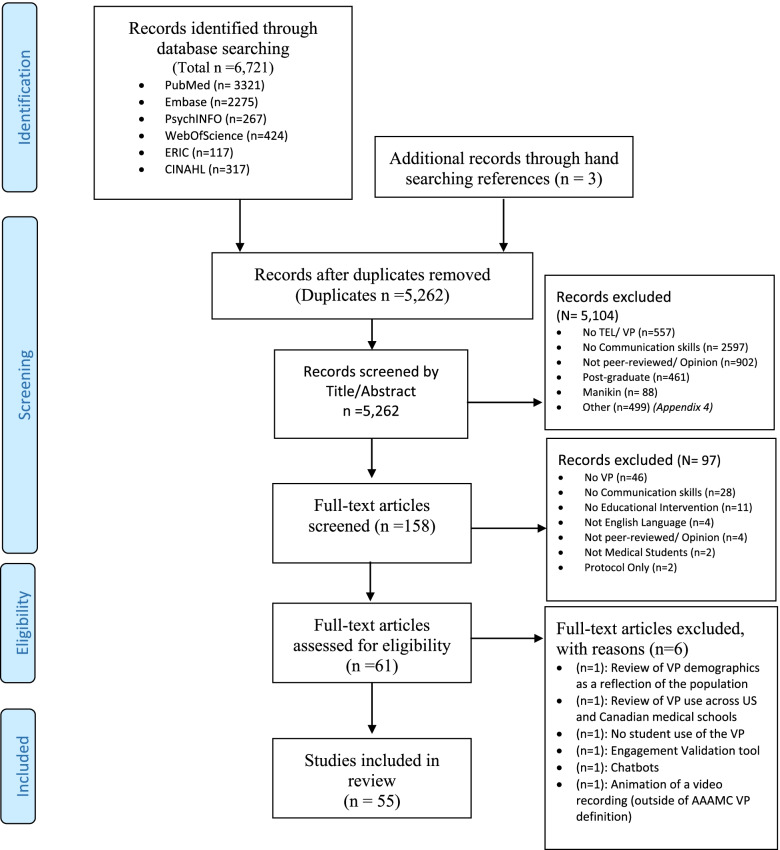
PRISMA flow chart

The search resulted in 5,262 references after de-duplication, of these we screened 158 full text articles, and 55 had sufficient data fulfilling inclusion in this scoping review (see PRISMA Fig. 1). Cohen’s kappa for selection was 0.76 and extraction was 0.84, denoting substantial agreement. The first article was published in 1976 with two later peaks; 2007 and 2018, papers originated from 16 countries by 44 unique authors. Fifty-five per cent of publications emerged from five main Journals (see Table 1).

**Table 1:** Results of data extraction (Part 1 of 2)

Results		% (n) No. of Total Papers
Region of Origin	Europe	47% (26)
	North America	40% (22)
	South America	2% (1)
	Asia	4% (2)
	Australia	7% (4)
Journal of Publication	BMC Medical Education	16% (9)
	Medical Teacher	13% (7)
	Stud. in Health Tech. Information	13% (7)
	J. of Med. Internet Research	7% (4)
	Academic Medicine	5% (3)
Learner Year Group	Senior (≧3^rd^ Year)	42% (23)
	Junior (1^st^-2^nd^ Year)	23% (13)
	Mixed Year Groups	11% (6)
	No report	24% (13)
No. of Learners	Learner no. Reported	90% (50)
	No learner no. Reported	10% (5)
Frequency of VP use	Once	31% (17)
	>1	29% (16)
	Variable per student	4% (2)
	Not reported	36% (19)
Duration of VP use	Reported	29% (16)
	Not Reported	71% (39)
Learning Outcome of VP	Communication Skills	40% (22)
	Clinical Reasoning Skills	47% (26)
	Empathy	5% (3)
	Non-verbal Communication	2% (1)
	Not stated	6% (3)
Kirkpatrick Evaluation	Level 1	40% (22)
	Level 2a	13% (7)
	Level 2b	33% (18)
	Level 3	0% (0)
	Level 4	0% (0)
	No Kirkpatrick Level discernible	14% (8)
Feedback, Reflection & Assessment	Feedback Provided	64% (35)
	No Feedback Reported	36% (20)
	Feedback During VP	16% (9)
	Feedback at End of VP	35% (19)
	No report on Feedback timing	49% (27)
	Formative Assessment Included	27% (15)
	Summative Assessment Included	13% (7)
	Self-Reflection Included	15% (8)
	No Assessment Reported	45% (25)

### The learners

Seventy six percent of core papers reported on learner year group with 42% (*n*=23) of studies including senior medical students, defined as above or in 3^rd^ year, 23% (*n*=13) included junior students (years 1-2), and 11% (*n*=6) included learners from more than one year-group. The median number of learners in the studies was 78 (Range 5 to 306), with 33% (*n*=18) comprising <50 learners, 18% (*n*=10) had 50-100 learners and 31% (*n*=17) included >100 learners. Eighteen per cent (*n*=10) of all studies did not report on the number of learners. The frequency of VP use was once in 31% (*n*=17) of papers, 29% (*n*=16) had >1 frequency of VP use, 4% (*n*=2) variable frequency of use, and 36% (*n*=19) of articles had no usage data. Of the 29% papers that reported duration of the VP intervention, the average was 44.90 minutes (range 10-120 minutes).

### Communication skills

The intended learning outcomes of VPs were communication skills in 40% (*n*=22) e.g. history taking, counselling skills, breaking-bad news, and inter-physician communication. Five per cent of studies (*n*=3) had their main learning outcome denoted specifically as empathic communication and 2% (*n*=1) as non-verbal communication skills. However, communication skills were often a component of VPs focussed on clinical reasoning skills in 47% (*n*=26) of papers. Further details on the use of VPs by medical specialty and case topic are provided in Table 3.

### Kirkpatrick evaluation

Eighty-five per cent of papers (*n*= 47) had a discernible Kirkpatrick level. The majority described Kirkpatrick level 1 (learner satisfaction or reaction, 40%, *n*=22), 13% (*n*=7) level 2a (attitude change), and 33% (*n*=18) Kirkpatrick level of 2b (change in knowledge/skills). No paper demonstrated a Kirkpatrick level of 3 or 4 (see Supplementary Appendix 2).

### Student evaluation

The majority of papers (73 %, *n*=40) included some element of student evaluation, reflecting a generally positive view of VP use, with one paper reporting an overall negative student evaluation because of perceived VP difficulty [34].

### Virtual patients

#### Framed activities

Thirty-five per cent (*n*=19) of papers included the VP within a mandatory blended learning course (see Table 2). The highest proportion of papers (47%; *n*=26) did not integrate the VP into existing curriculum, with one paper reporting a failure to achieve intended curricular integration [35].

**Table 2:** Results of data extraction informed by the Ellaway & Davies Framework (Part 2 of 2)

Results		% (n) No. of Total Papers
Framed Activities	Curricular Integration	35% (19)
	Not Integrated into Curriculum	47% (26)
	Not reported	18% (10)
Constructed Activities	Independent Use	73% (40)
	Used in peer group	9% (5)
	With Tutor assistance	4% (2)
	Not reported	15% (8)
Encoded Activities (Operational Type)	Type-in text	36% (20)
	Selected Responses	22% (12)
	Voice command	7% (4)
	Gestures (e.g. body, facial)	4% (2)
	No data on operation type	31% (17)
Encoded Activities (Media)	Human Avatar	24% (13)
	Static Image	15% (8)
	Non-specific Multimedia	11% (6)
	Text-only	2% (1)
	Media not reported	40% (22)
Encoded Activities (Sequencing)	Branching Design	16% (9)
	Linear Design	9% (5)
	Sequencing not reported	67% (37)

**Table 3:** Uses of VPs for communication skills in undergraduate medicine by specialty and topic. A number of papers presented VPs with more than one topic.

Task	Specialty	Topic	No. of Papers
Communication Skills	General	Anxious/ aggressive patient	*n*= 6*
		Patient ignoring doctor’s advice	*n*= 1*
		Frequent demander	*n*= 1*
		Interprofessional Communication	*n*= 1*
		Breaking Bad News	*n*= 3*
		Non-verbal Communication	*n*= 1
		Empathy	*n*= 3
		Not Specified	*n*= 10*
	Medicine	Abdominal Pain	*n*= 3
	Surgery	Back Pain	*n*= 2
	Paediatrics	Ear complaint	*n*= 1
		Asthma	*n*= 1*
		Gastroenteritis	*n*= 1*
		Cultural differences	*n*= 1*
	Psychiatry	Depression	*n*= 1
		Bulimia nervosa	*n*= 1
Clinical Reasoning	General	Not specified	*n*= 8*
	Medicine	Renal Failure	*n*= 2*
		Haematology	*n*= 3
		Cardiology	*n*= 5*
		Recurrence of TB	n= 1*
		Neurological disease	*n*= 1
		Community Acquired Pneumonia	*n*= 1*
		Neuro-musculoskeletal	*n*= 1*
		Oncology	*n*= 1
	Surgery	Chronic Sub-dural Haematoma	*n*= 1*
		Surgical OSCE	*n*= 1
		Spinal Trauma	*n*= 1
		Colon Cancer	*n*= 1*
		Renal Stenosis	*n*= 1*
		Abdominal Pain	*n*= 3
	General Practice	Safe to exercise	*n*= 1

*denotes the counts where the topic was associated with another case within the same paper

#### Constructed Activities

The majority of VPs were used independently by students (73%, *n*=40), 13% (*n*=7) of VPs were used in peer groups or with tutor assistance and 15% (*n*=8) of articles did not provide information.

#### Encoded Activities

VPs can be presented to the learner with a multiplicity of media, functionality and sequencing. In order to navigate through the computer program, most papers used VPs with type-in-text (36%, *n*=20), followed by selected responses (22%, *n*=12), voice-command (7%, *n*=4), and gestures (4%, *n*=2). There was no data on encoded activity in 31% of papers (*n*=17) (see Table 2).

Media representation was human avatars in 24% (*n*=13), static images (15%, *n*=8), ‘multimedia’ unspecified (11%, *n*=6) and text-only (2%, *n*=1) [36], 40% (*n*=22) of papers did not report on media used for VP.

A branching design was used in 16% (*n*=9), 9% (*n*=5) used a linear design, and 7% (*n*=4) had a mixture of designs. The VP sequencing was not reported in the majority of papers (67%, *n*=37).

### Feedback, reflection and assessment

Feedback was provided with the VP educational intervention in 64% (*n*= 35) of papers, with 29% (*n*=16) providing learner question analysis or comparison to expert performance, 15% (*n*=8) providing a scoring rubric e.g. empathy score, and 9% (*n*=5) a variety of other feedback methods e.g. tutor-led debrief, expert feedback, peer feedback, standardized patient feedback (see Table 1). Eleven per cent (*n*=6) of papers mentioned feedback but did not provide details of format. Feedback timing was reported in 51% (*n*=28) of papers, with 16% of VPs (*n*=9) providing feedback during the intervention and 35% (*n*=19) at the end of the intervention. Only 15% of papers (*n*=8) used student self-reflection as part of the VP educational intervention. 26 papers (47%) included an assessment following the VP intervention: 27% (*n*=15) used formative assessment methods and 13% (*n*=7) included summative assessment.

### Cost

Eleven per cent (*n*=6) of articles included cost analysis, with wide variability in reporting. One study reported total costs of <$7000 per prototype system [37], another used cost per hour at $12/hour [38] for technology development, and another at $15/hr/student [39]. One study performed a detailed cost analysis computing the monthly average costs to develop and maintain a VP at $324.75 [35]. Several other studies reported the VP system to be ‘expensive’ without detailed cost analysis (*n*=6, 11%). The majority of articles however did not report or comment on cost.

## Discussion

This review demonstrates current approaches to the use of virtual patients in developing consultation skills for medical students and given resource implications, the pragmatic analytical lens used highlights areas for potential development. Students value VP interactions, and are especially positive about appropriately sequenced authentic VPs (although they can be negative if overwhelmed). A holistic consultation approach is taken with most VPs, supporting clinical reasoning and communication skills development. However, one-off interactions with VPs are plentiful, which seems to run counter to the potential of VPs for individual repeated practice and mastery learning. Similarly, the individual growth opportunity for feedback and reflection was relatively under-utilised.

## Comparison with previous research

A recent systematic review of digital education for communication skills used a broad definition of ‘digital tools’ synthesised evidence from 12 RCTs [17]. It found a high degree of heterogeneity in the literature, a paucity of a common language, and unclear theoretical underpinnings [17]. It concluded that digital tools could be as effective as traditional methods of instruction. While our review supports many of these findings, we focussed on ‘virtual patients’, and included a broader range of evidence to reflect the diversity of medical education approaches. Our findings suggest that it is not only the tool itself (VPs) but also the educational activities surrounding the VP that influence learning outcomes [40].

Other reviews have evaluated all VP applications [23, 40, 41] or reviewed an interlaced feature of communication skills such as empathy [42] or clinical reasoning skills [43]. This scoping review advances the field by outlining the breadth of research within a systematic and educationally informed framework, focussed specifically on VP tools and surrounding educational activities, for the key development of medical student communication skills. In doing this we provide a clear and structured overview of gaps in the literature (see Table 1 & 2), some of which have been remarkably consistent over time and which future investigation should address.

## Kirkpatrick framework & student evaluation

Student acceptance and evaluation are important for engagement, motivation and learning [35]. Students had an overall positive view of VPs (Kirkpatrick Level 1), citing high levels of engagement, feeling less pressure and anxiety compared to ‘real patient’ interactions, being able to practice without the fear of making a mistake, and so seeing VPs as a valuable learning experience [44–48]. Further, it was found that students value authenticity both in the patient presentation and interaction [26, 48–50].

Students felt negatively about VPs when they perceived them to be unrealistic, limited in natural responses, repetitive or the task was too difficult [34, 51]. Limitations in VP technology incited learner frustration, particularly if computer literacy was low amongst users, the technology was slow or did not understand the students’ phrasing [35, 47]. It is important for educators to consider that technological advancements can leave students behind, as not all students will adapt fluidly to e-learning [52]. We identified emerging formats of VPs e.g. voice-activation with natural conversation, which may become the new frontier of digital communication skills training to enhance authenticity, this could provide useful modelling for students for whom English is a second language or who transition globally to different work environments, although it could disadvantage those learners with speech or learning impairments [53].

### Activity theory framework for VPs

#### Framed Activities

Framed activities refer to how the VP sits in the wider curriculum. One study integrated VP educational tools for both teaching and assessment, and found that students who developed their skills with a Web-based SP had superior exam results compared to those offered in the traditional curriculum [54]. Despite this, our findings do not reflect widespread curricular integration as the majority of research was conducted as a pilot study or an opt-in module for students. Unfortunately, this somewhat piecemeal approach leads to a disassociation between the intended learning outcomes, teaching, and assessments, not supporting the learner to develop a holistic deeper understanding [55, 56]. Notably the earliest study in our scoping review (O’Neil, 1976) had proposed future research to include VP curricular integration to enhance outcomes [35] that our findings also support.

#### Constructed Activities

Constructed activities refer to how teachers and learners make use of the artefact, e.g. its application, frequency and mode of use. Our review identified broader clinical reasoning skills as the main application of VPs, and fewer studies evaluated communication skills alone. This focus on clinical reasoning skills upholds others’ views (Cook & Triola [14, 40] and Ellaway [57]) that the VP’s forte lies in the development of holistic clinical reasoning skills. Seemingly, there was a brief decline in studies published on communication skills training with VPs, before regaining some momentum in recent years. One study found that VP interventions improved communication skills compared to traditional methods [58]. This is a particularly important field for educators seeking alternative computer-based solutions to increased class sizes, reduced access to patients, clinical sites and economic pressures, all now compounded by the Covid-19 pandemic.

VPs offer ample opportunity for mastery-learning and repeated practice until competency is achieved [6], the novice learning through experience and exposure to a large number of cases [40]. Curiously, this scoping review identified that the majority of articles described a one-off educational intervention, without repeated practice. Mastery learning theory regards time spent on skill development as an inadequate marker of competency [6, 19]. However, one study found that longer time spent using the VP resulted in higher test scores [59]. Another study assessing clinical reasoning development, found that the students given a single case of longer duration, had a non-statistically significant but numerical benefit over those students who had a limited duration of exposure but higher volume of VP cases in the same timeframe [34]. Students themselves reported a negative view and increased anxiety from time-constraints imposed on VP interactions [34, 60]. Our scoping review highlights that educators need to consider the frequency and duration of VPs and its impact on communication skills acquisition. VPs inherently offer the advantage of flexibility, as students can re-visit and practice at their individual learner-set pace [18]. We propose that students should be provided with maximal opportunity to draw out VP’s full learning potential by repeated practice.

### Encoded activities

Encoded activities refer to technical design; how the VP is presented (media), how the learner navigates through the program (operation) and how the VP behaves based on the student selection (sequence). Most VPs used either a static or an animated representation of a patient, and occasionally pre-recorded video clips of either SPs or real patient responses. One study used text-only without a patient image [36] whereas in contrast several studies used life-sized projections of avatars. One study found that the larger screen improved learner’s emotional engagement and immersion level, important for skill acquisition [37, 48, 61–63]. In some studies, students felt that VP responses were unrealistic; the VP’s natural language processing or recognition was not able to ‘understand’ the student or curtailed the ability of the student to express themselves due to the limitation of technology [47, 48, 64]. Natural language processing advances have improved VP responses. Learners cautioned against ‘hyper- dynamic’ VPs that may lead to cognitive overload and distraction [26, 43]. From our review we recommend educators to balance computerized authenticity with instructional design.

Sequencing within a VP can be branching or linear. Linear design follows a pre-defined sequence to a single end-point. In branching models, there are a number of alternative pathways with different end-points [26]. Bearman et al [64] studied the ‘problem-based design’ and ‘narrative design’ in the context of communication skills, and concluded that there was a significant difference favouring the narrative approach for the development of communication skills training. It is clear that the encoded activities of the VP influence learner outcomes. Surprisingly, there is low reporting on the sequence design of VPs in this literature (33%) which should be considered by future researchers when evaluating effective design for communication skills acquisition.

### Feedback & reflection

Feedback and reflection are vital learning tools in clinical education [65]. Our review showed that the students valued feedback when it was received [66]. One study found that students that received feedback had better outcomes, superseding the mode of teaching [67]. This scoping review found that there was low reporting on details of feedback such as the type of feedback and timing of feedback. Also relevant is reflection, giving learners opportunity to assimilate skills and concepts, promoting individual growth [65].Very few studies incorporated occasion for self-reflection and therefore overlooked this valuable learning opportunity.

### Cost

Our review highlights commentary stating that VP development and maintenance is expensive: both in monetary and human resource terms. However, only a limited number of studies have reported on cost, which has important contemporary significance, in any detail [35, 38, 39]. One study evaluated VP use in a resource-limited country, and interestingly found it to be a viable option, although sadly researchers did not share cost details [68]. Currently the pandemic has precipitated an urgent pivot to technology. Bearing in mind the not insignificant resource implications of development, we recommend filling this gap by explicitly attending to, and reporting the financial and human resource impact in the development of virtual patient tools. Furthermore, it seems that researchers cannot truly comment on cost-effectiveness until the VP is constructively aligned and integrated into the curriculum [69].

### Limitations

This scoping review only included studies reporting on undergraduate medical students, we cannot therefore comment on the generalizability of these findings for other areas of health professions education. A myriad of terms can be used for ‘virtual patients’ and ‘communication skills’, although due process with testing multiple iterations of the search terms was undertaken with the help of a specialist librarian, it is possible that, despite these precautions and testing, some relevant synonymous terms may have been omitted, leading to potential omissions of eligible studies. The risk of this was mitigated by hand searching the references of included studies. Moreover, this area of research is complex and emerging, making synthesis of information of such heterogeneous evidence more challenging. Definitional rigour would assist future evaluation and development. No analysis of quality was undertaken due to the nature of scoping reviews, therefore poor quality studies could potentially have been included in the review.

## Conclusion

This scoping review is pragmatically informed by activity theory [32] focussed on the affordance of virtual patients for medical student learning. We found that VP educational interventions for communication skills are in need of a common language, more detailed reporting (especially of educational consequences and resource implications), and instructionally informed methodology. Potential for full curricular integration, blended learning and repeated practice seems to be under-utilised. Research in this area remains relatively small, and incomplete: it needs to be supported by embracing both definitional and methodological challenges. COVID-19 has thrown medical education into a virtual world an element of which is likely to stay and so the learning potential of VPs should be maximised. To advance the field we recommend educationalists to evaluate not only the tool itself, but how it aligns with its surrounding educational activities, and the affordance for learning. In an era where educationalists are faced with mounting challenges, augmented by the Covid-19 pandemic, there has never been a more important time for insights into competency development for communication skills on a Virtual Patient platform.

## Supplementary Information

**Additional file 1:** Supplementary Appendix 1, Appendix 2, Appendix 3, Appendix 4.

## Data Availability

Not applicable

## Abbreviations

VP: Virtual Patients
AAMC: American Association of Medical Colleges
CBE: Competency Based Medical Education
TEL: Technology Enhanced Learning
STORIES: STructured apprOach to the Reporting In healthcare education of Evidence Synthesis
BEME: Best Evidence Medical Education
PRIMSA: Preferred Reporting Items for Systematic Reviews and Meta-Analyses
