# Supplementary material for: A scoping review: virtual patients for communication skills in medical undergraduates
Source: BMC Med Educ. 2022 Jun 3;22:429. doi: 10.1186/s12909-022-03474-9 (PMC9166208; doi:10.1186/s12909-022-03474-9)
Supplement: Supplementary file 1 — Additional file 1: Supplementary Appendix 1, Appendix 2, Appendix 3, Appendix 4. [file 12909_2022_3474_MOESM1_ESM.docx]

Supplementary Appendices

| Step 1: Identifying the research question | Determination of a research question that provides breadth of coverage and serves as a roadmap for later stages. |
| --- | --- |
| Step 2: Identifying the relevant studies | Determination of the sources, time span, language and search terms for the literature search. |
| Step 3: Study Selection | Development of inclusion and exclusion criteria through an iterative process. |
| Step 4: Charting the Data | Development of a data charting form for extracting the relevant study information. |
| Step 5: Collating summarising and reporting results | Structuring and reporting the extracted data based on numerical and thematic analysis. |
| Step 6 (Optional): Consultation | Involvement of key stakeholders to arrive at insights beyond those in the literature e.g. students and educators |

**Supplementary Appendix 1:** Arksey & O’Malley Framework for scoping review methodology **^26^**.

| **Kirkpatrick Level** | | **Description** |
| --- | --- | --- |
| **Level 1** | Participation | Covers learner’s views on the learning experience, its organization, presentation, content, teaching methods and aspects of instructional organization. |
| **Level 2a** | Attitudes/ Perceptions | Outcomes that relate to changes in the reciprocal attitudes or perceptions of participants towards the intervention. |
| **Level 2b** | Knowledge/Skills | Modification of the acquisition of concepts, procedures and principles or the acquisition of thinking/problem-solving psychomotor and social skills. |
| **Level 3** | Behavior Change | Evidence of the transfer of learning to the workplace or willingness of learners to apply new knowledge or and skills. |
| **Level 4a** | Organizational Outcomes | Wider change in the organization or delivery of care attributable to the educational program. |
| **Level 4b** | Patient Outcomes | Any improvement in the health and well-being of patients/clients as a direct result of an educational program. |

**Supplementary Appendix 2:** Kirkpatrick evaluation levels adopted from Yardley & Dornan (2011) **^28^**, originally based on the BEME specimen coding sheet.

**
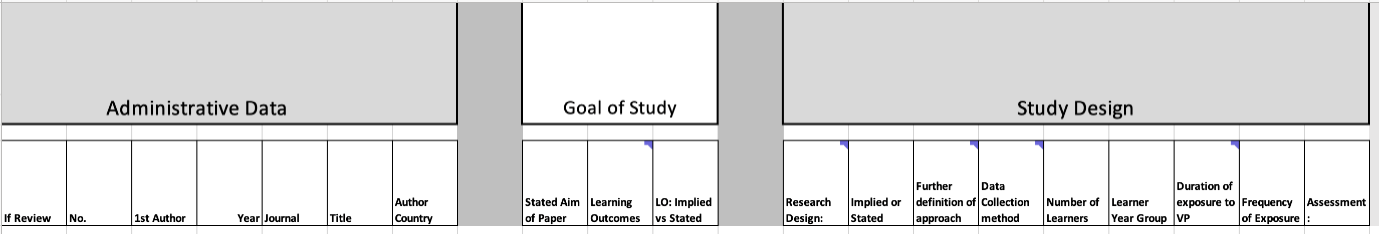
**

**
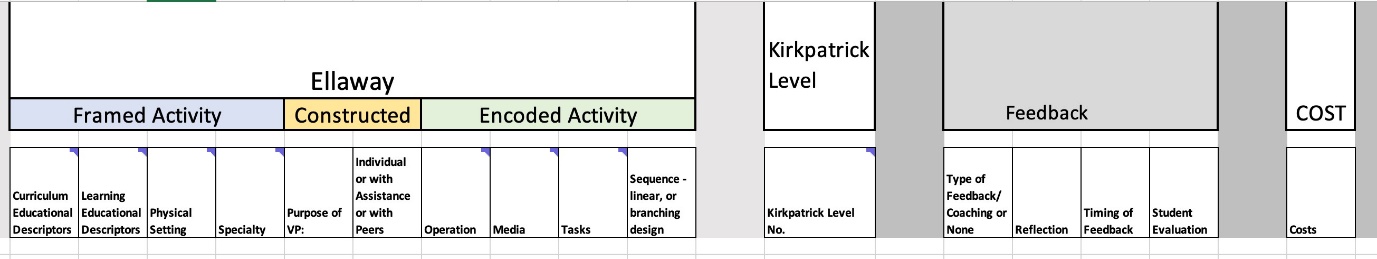
**

**Supplementary Appendix 3:** Data abstraction form.

| Exclusion Criteria | No. of Records |
| --- | --- |
| Not English Language | n= 6 |
| Interprofessional Education | n= 158 |
| Patient Information/ Patient Education | n= 18 |
| Not Healthcare related | n= 10 |
| No Educational Intervention | n= 25 |
| Other Healthcare Professionals (not medical) | n= 273 |
| Social Media | n =9 |

**Supplementary Appendix 4:** Other Exclusion Criteria from PRISMA flow chart
